# Effects of observing own/others hand movement in different perspectives on mu rhythm suppression: an EEG study

**DOI:** 10.1186/s40101-024-00369-0

**Published:** 2024-09-04

**Authors:** Nakyeong Shin, Yuki Ikeda, Yuki Motomura, Shigekazu Higuchi

**Affiliations:** 1 Graduate School of Integrated Frontier Sciences, Kyushu University, 4-9-1 Shiobaru, Minami-Ku, Fukuoka City, Fukuoka, Japan; 2 Faculty of Health Science, Kyorin University, 6-20-2 Shinkawa, Mitaka-Shi, Tokyo, Japan; 3 Faculty of Design, Kyushu University, 4-9-1 Shiobaru, Minami-Ku Fukuoka City, Fukuoka, Japan; 4 Research Fellow of the Japan Society for the Promotion of Science, Kojimachi Business Center, Building, 5-3-1 Kojimachi, Chiyoda-Ku, Tokyo, Japan

## Abstract

**Background:**

Previous studies have reported that the sense of “self” is associated with specific brain regions and neural network activities. In addition, the mirror system, which functions when executing or observing an action, might contribute to differentiating the self from others and form the basis of the sense of self as a fundamental physical representation. This study investigated whether differences in mu suppression, an indicator of mirror system activity, reflect cognitions related to self-other discrimination.

**Methods:**

The participants were 30 of healthy college students. The participants observed short video clips of hand movements performed by themselves or actors from two perspectives (i.e., first-person and third-person). The electroencephalogram (EEG) mu rhythm (8–13 Hz) was measured during video observation as an index of mirror neuron system activity. EEG activity related to self-detection was analyzed using participants’ hand movements as self-relevant stimuli.

**Results:**

The results showed that mu suppression in the 8–13-Hz range exhibited perspective-dependent responses to self/other stimuli. There was a significant self-oriented mu suppression response in the first-person perspective. However, the study found no significant response orientation in the third-person perspective. The results suggest that mirror system activity may involve self-other discrimination differently depending on the perspective.

**Conclusions:**

In summary, this study examined the mirror system’s activity for self and others using the EEG’s mu suppression. As a result, it was suggested that differences in self and others or perspectives may influence mu suppression.

**Supplementary Information:**

The online version contains supplementary material available at 10.1186/s40101-024-00369-0.

## Background

Understanding the distinctions between self and others, and the intrinsic sense that our body and consciousness are distinct from those of others, is a fundamental aspect of human cognition and adaptability. This distinction is crucial for effective social interaction and communication, which are vital for human survival and thriving within complex social structures. Previous studies have indicated that disruptions in the sense of self that cause difficulties in interacting with others and society are related to mental illnesses [1, 2], suggesting that the sense of distinction between the self and others significantly influences communicating with surrounding people. Neuroscientific investigations into the mechanisms of the sense of self have pointed to several key brain regions around the cortical midline. These include the orbital and adjacent medial prefrontal cortex, the dorsomedial prefrontal cortex, and the anterior and posterior cingulate cortex, which are thought to play vital roles in self-referential processing [3, 4]. Additionally, collaborates with these cortical midline structures to form the neural basis of the sense of self [5].

The mirror neuron system (mirror system) is a neural network that functions when executing or observing an action. Functional magnetic resonance imaging (fMRI) studies have confirmed that this system consists primarily of a network of sensorimotor cortical areas, including the inferior parietal lobule, inferior frontal gyrus, and adjacent ventral premotor areas [6]. The mirror system is involved in understanding and imitating the actions of others and is thought to facilitate social learning and communication by enabling individuals to simulate and understand the intentions and emotions of others [7, 8]. The mirror system’s activity can be examined by using electroencephalography (EEG). Previous studies have indicated that the mu rhythm, defined as the 8–13 Hz rhythm arising around the brain’s central sulcus at rest, is suppressed when executing an action. Other studies confirmed that mu suppression also reflects action simulation [9–11]. Moreover, mu suppression occurs when observing an action. Therefore, it has often been used as an effective mirror system activity index, although its validity is controversial [12–14]. In addition, studies have reported a functional dissociation within the lower (8–10 Hz) and the upper (11–13 Hz) mu suppression bands. For example, one study reported that the lower mu is related to a widespread EEG pattern, non-specific to movement types. In contrast, the upper mu shows a more focused, movement-type-specific pattern, which is different for finger and foot movements [15]. Furthermore, the inequality between lower and upper mu suppression changes when observing and executing an action [16, 17]. However, it remains unclear whether these differences are related to the distinctions between self and others.

While assimilation of self and others is effective in imitation and observational learning, the ability to discriminate between self and others is equally important for an individual’s social adaptation and harmony with the environment, and the mirror system contributes to both. The mirror system reacts to own and others’ actions, seemingly assimilating self and others in brain representations. However, previous fMRI studies have indicated that the mirror system’s component regions, mainly in the right hemisphere, selectively responds to self-related stimuli, including face [18, 19], hands [20], and voice [21, 22]. The researchers suggested a specific role for the mirror neuron network in self-other discrimination. In motor or simulation theories, perception is thought to occur through motor simulation or a mapping of the actions of others onto one’s motor system. The mirror system regions may contribute to the construction of communication between individuals via simulation mechanisms that map the actions of others onto their motor repertoires. It is speculated that mirror system regions such as the inferior parietal lobule and inferior frontal gyrus are more strongly activated to one’s image or self-related stimuli because of the ease with which one can map oneself onto one’s motor system when comparing the self to an external stimulus that is most like oneself. Molnar-Szakacs and Uddin proposed a model of the conceptual sense of self-other when responding to social cognitive demands, in which there is an interaction between the network responsible for higher-order mentalizing and the network responsible for embodiment, including the mirror system [23, 24]. This model describes the relationship between empathy as an inference of another’s mental state and the mirror system as an embodied simulation. However, the mirror system’s contribution to brain representations of the self and others remains to be examined.

When selecting a method to investigate the relationship between the sense of self and the mirror system, it is critical to examine whether mu suppression reflects brain processing of self-other discrimination. The mu suppression when observing hand movements has been examined in detail because mirror system activity is associated with motor action, including the effects of movement types [25–27] and perspectives [28–30]. For instance, Angelini et al. examined brain activity when observing hand movements from four perspectives [30]. They demonstrated that the most robust mu suppression occurs when observing movements from the first-person perspective compared to other perspectives, perhaps because first-person hand movements are more likely to generate the sensation of belonging to the self than a third-person movement. As a result, specific studies have suggested that mu suppression in the first-person perspective is a response to self-related stimuli. However, only a few studies have investigated the validity of the contention that perspective differences represent differences between the self and others. Therefore, the present study investigated the effects of differences between the self and others on mu power fluctuations by using the participants’ hand movements as a self-related stimulus that produces self-other discriminations in practice. Nagai and Tanaka demonstrated that observing the action of the own and others’ hands produces more robust mu suppression for the own hand [31]. However, their study was limited to the first-person perspective. Hence, the participants in the current study conducted self-other discrimination from first- and third-person perspectives. We predicted that mu suppression to the own hand would increase under the first- and third-person perspectives if mu suppression reactivity were affected by the hand’s owner regardless of the perspective. Therefore, this study investigated the extent to which mu suppression reflects differences between self and others.

## Methods

### Participants

Healthy college students (*N* = 30, 8 women; mean age = 23.58 ± 1.59 years) participated in this study. All the participants were right-handed, defined as a laterality quotient of 40 or higher in the 10-item Edinburgh Handedness Inventory. The participants gave their written informed consent for participating in this study before the experiment. The Ethics Committee of Kyushu University approved the study. We excluded the data of five participants from the analysis: two because of excessive and continuous motion artifacts at the measurement and three because the number of trials remaining after removing trials in the EEG processing process was less than the required number (≤ 20 trials per condition).

### Equipment

The study was conducted in an acoustically and electrically sealed room. Stimuli were delivered with Presentation Ver. 18.2 (NBS Inc.) and an LCD (RL2460-B, BenQ Corp.) refreshing on 60 Hz. The display was placed 60 cm in front of participants. EEG was recorded by using a 64-channel EEG System (Net Amps 200, Electrical Geodesics Inc.) and a sensor net (Hydrocel Geodesics Sensor Net, EGI) with acquisition software (Net Station 4.3.1, EGI).

### Stimuli

**Fig. 1:**
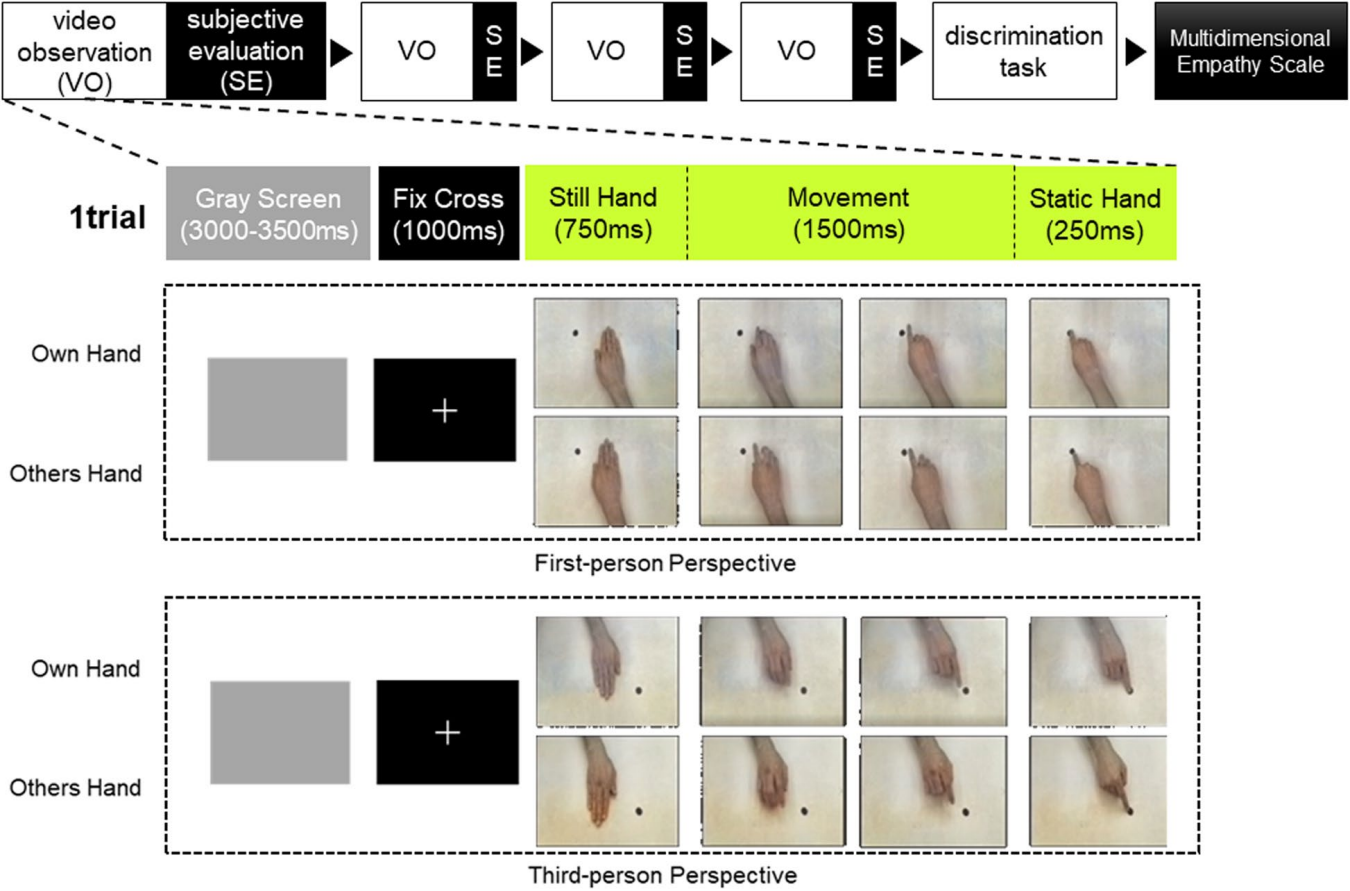
Experimental protocol, conditions, and stimuli

The stimuli consisted of video clips (each clip 2500 ms in length) showing right-hand movements that we presented from two perspectives (Fig. 1). Two actors, a man and a woman, performed the action in half the video clips. The participants performed the action in the other half. Before the experiment, the following simple hand movements of the participants were recorded and edited as stimuli: pointing to a black dot on a table and clenching hand. A metronome controlled the movement’s speed. Two cameras, one facing the other at an identical height, recorded the action. Each video clip comprised the following sequence: the Still Hand (750 ms), a black dot and the hand in a resting position; movement (1500 ms); static end (250 ms), and the static hand in the final position. The experiment comprised four conditions: 2 hand owners × 2 perspectives: (1) own hand in the first-person perspective (egocentric), (2) others’ hand in the first-person perspective, (3) own hand in the third-person perspective (allocentric), and (4) others’ hand in the third-person perspective.

### Experimental procedure

The participants sat facing a monitor in front of them. We fitted an EEG cap on the participants’ heads and instructed them to observe the video with their right hand relaxing on a mouse. The experimental procedure included 120 trials (30 for each condition × 4 conditions) presented in four blocks. As shown in Fig. 1, each trial included (1) a gray screen (presented between 3000 and 3500 ms), (2) a central fixation cross (presented for 1000 ms), and (3) the video clip (presented for 2500 ms). Each block contained the clips seen from only one perspective (egocentric or allocentric). We fully randomized the blocks and trial sequences and asked the participants to respond to randomly presented image trials showing the still hand resting by clicking the mouse. These image trials appeared three to six times in the blocks we excluded from the subsequent analysis. After each block, participants responded to a questionnaire assessing how well they could distinguish themselves from others using a 5-point scale (subjective evaluation), and we also assessed the participants’ sleepiness degree using the Karolinska Sleepiness Scale (KSS). We did not ask the participants to discriminate between the self and others during the video observation trials to avoid artifacts. However, we confirmed they could discriminate between themselves and others by asking them to review all the video clips and classify them as a self or others discrimination task after all video observation blocks. The participants also completed the Multidimensional Empathy Scale (MES) designed to discriminate between self and other orientations of cognitive and emotional components. The MES consists of five subscales: other-oriented emotional reactivity, self-oriented emotional reactivity, emotional susceptibility, perspective-taking, and fantasy. We planned to use the MES results for a different study that will examine the relationship between mu suppression and empathic characteristics shown in previous study [32].

### EEG data acquisition and analysis

**Fig. 2:**
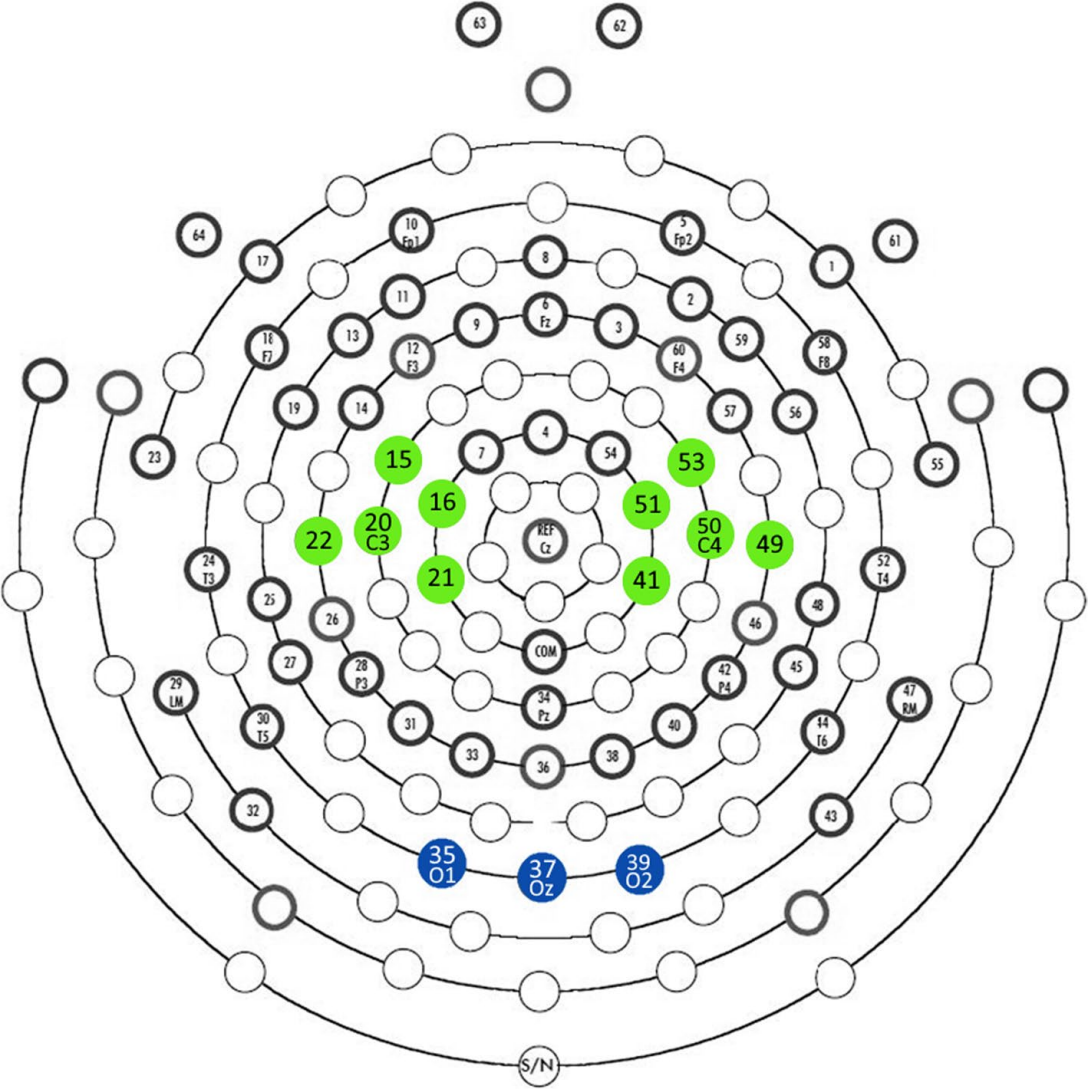
Clusters of electrodes were used for statistical analyses. The central cluster of the 64-channel array is a grouping of electrodes around C3 and C4 (marked green). The occipital cluster is a grouping of electrodes adjacent to Oz (marked blue)

**Fig. 3:**
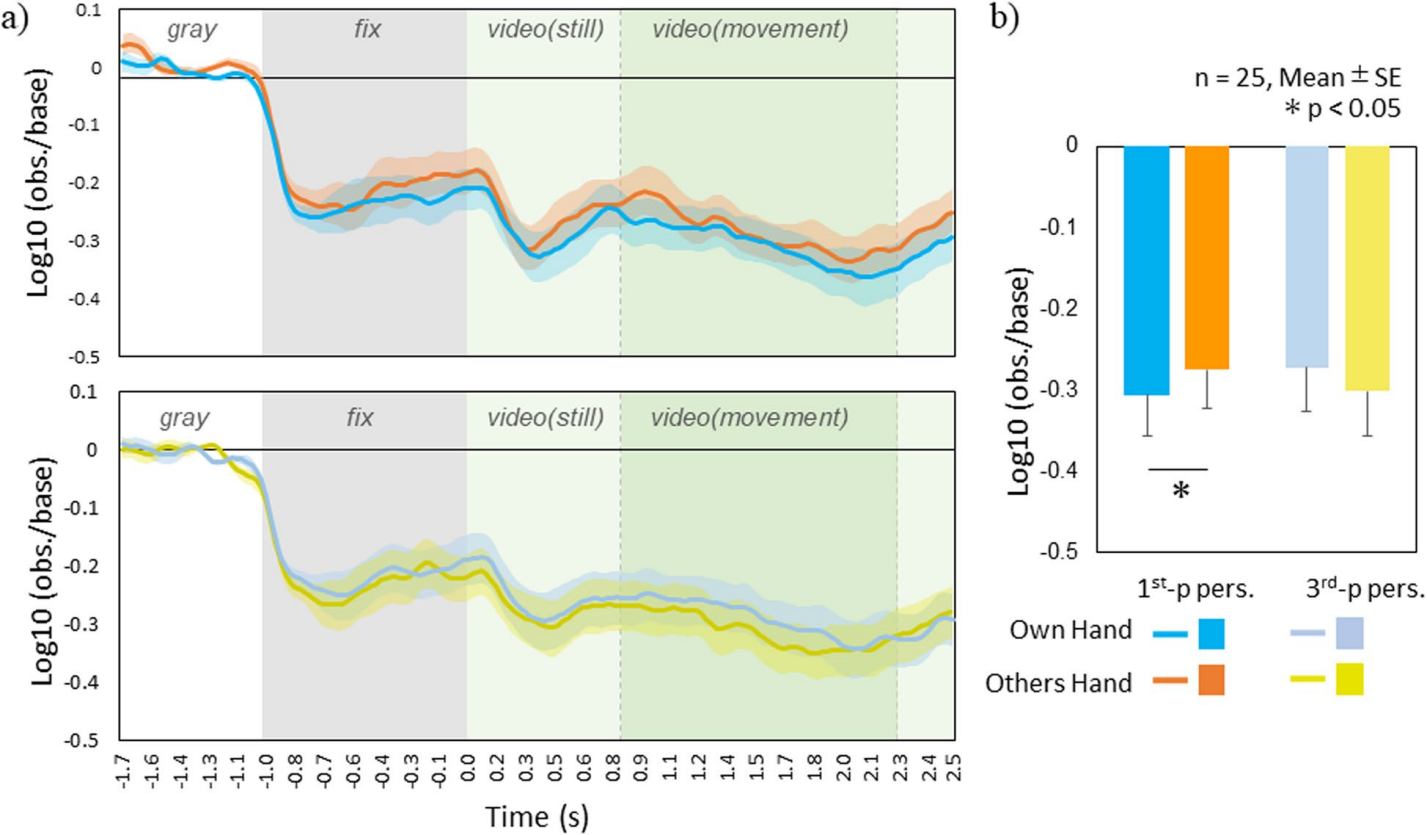
Mu (8–13 Hz) power suppression. **a** The log10 power ratio’s time course for each condition. The elements within an epoch (gray background as the baseline, the fixation cross, and the video clip including still and moving parts) are labeled in different colors. **b** Mean (-SE) log10 power ratio when observing the moving part of video clips under different conditions. The graph represents the significant hand owner × perspective interaction in the 2 × 2 repeated measures ANOVA. Asterisks indicate a significant difference among perspectives

We recorded 64 channels of EEG at a sampling rate of 500 Hz (0.01 Hz high-pass filter) with a vertex reference. The impedance of the electrodes was maintained below 60 kΩ. Offline analyses were conducted using MATLAB (MathWorks, Inc.) and EEGLAB toolboxes [33]. The EEG data were filtered (1–35 Hz) and segmented into single-trial epochs of 4500 ms. Each epoch consisted of three segments: (1) a gray screen presented before the fixation cross for 1000 ms, (2) the fixation cross displayed for 1000 ms, and (3) the video clip lasting 2500 ms. We used independent component analysis (ICA) to remove ocular, cardiac, and muscular artifacts from the EEG data. Time-frequency transforms were computed for each electrode in the 5 to 32 Hz frequency range for each cleaned epoch using a Morlet wavelet. Spectral data were baseline-corrected to account for individual variability in overall EEG power. Based on previous studies [26, 30], we used a 300-ms pre-stimulus period within the gray screen (from − 1650 to − 1350 ms before video clip onset) as the baseline. We baseline-corrected the spectral data obtained from each participant and condition by dividing the value of each time-frequency point by the mean spectral power of the baseline at the same frequency. Based on the literature [30], we used the central cluster around C3 and C4 for mu rhythm analyses, as shown in Fig. 2, and selected the lower (8–10 Hz) and upper (11–13 Hz) alpha bands. We averaged the ratio data across participants, conditions, and the left and right central clusters of electrodes (Fig. 3a) based on the evidence that mu rhythm subcomponents may have different functional properties. Then, we averaged the data from the movement section of the video clip (1500 ms, Fig. 3b). The still section was excluded from the average. Before statistical analysis, we applied a log10 transformation to each absolute power ratio value. A zero log10 ratio value indicated no EEG modulation, whereas a negative log10 ratio indicated EEG power suppression. We statistically analyzed the mean powers of each cluster as the dependent variables using a within-subjects, three-way repeated measures analysis of variance (ANOVA) with the band (lower, upper), hand owner (self, others), and perspective (first-person, third-person) as the independent variables. After a significant primary interaction, we conducted the Sequentially Rejective Bonferroni-corrected post hoc test.

## Complementary analyses

The occipital cluster (around Oz; Fig. 2) was used to check for the possible influence of volume conduction from visual cortices on the alpha-band range. As a result, there were no differences between conditions in occipital alpha suppression, confirming the possibility that the results of this study reflect the activity and characteristics of central regions. Please see “Occipital alpha suppression” in the Supplementary Information for further details of the occipital cluster’s results. There was no between-condition difference.

## Results

The mean correct response rate for the self-other discrimination tasks conducted after completing the observation tasks was 95%, confirming that participants could accurately distinguish their hands from another person’s (Table 1).

**Table 1:** Mean accuracy (standard errors) of own/others’ hand recognition

	1st-p pers	3rd-p.pers
Own hand	1.00 (0.00)	0.98 (0.08)
Other hand	0.95 (0.12)	0.91 (0.19)
Overall	0.95 (0.09)	

### Mu suppression

Results indicated a significant main effect of band (*F*(1,24) = 7.099, *p* = 0.013, *η*_*p*_^*2*^ = 0.23), showing an overall more potent suppression of lower mu. There were no significant main effects for hand owner (*F*(1,24) = 0.0062, *p* = 0.94, *η*_*p*_^*2*^ = 0.0003) or perspective (*F*(1,24) = 0.062, *p* = 0.80, *η*_*p*_^*2*^ = 0.0026). Additionally, there were no significant interactions involving band, including the three-way interaction between band, hand owner, and perspective (*F*(1,24) = 0.36, *p* = 0.55, ηp2 = 0.015).

There was a significant interaction between hand owner and perspective (*F*(1,24) = 4.82, *p* = 0.038, *η*_*p*_^*2*^ = 0.17). Post hoc analyses of the interaction showed that the simple main effect of hand owner was significant in the first-person perspective (*F*(1,24) = 4.52, *p* = 0.044, *η*_*p*_^*2*^ = 0.16), with mu suppression for the own hand being more potent than for the others’ hand in the first-person perspective (Fig. 3b).

## Discussion

This study examined whether mu suppression, indicating the brain’s information processing about the self and the mirror system’s activity, reflected differences in cognitions associated with self-other discrimination. Several studies have examined the relationship between mu suppression and observing action, including the first-person perspective as the self-viewpoint. However, few studies have investigated the hypothesis that perspective differences represent differences between the self and others. We investigated EEG activity related to self-detection using participants’ hand movements as self-relevant stimuli and examined the perspective-dependent mu suppression. The study analyzed mu suppression in the lower- and upper-frequency bands and examined differences based on hand ownership and perspective. The results did not indicate a significant interaction between the three factors, band, hand owner, and perspective, which precluded conclusions about the band-specific effects of self-other discrimination. However, a significant interaction between hand owner and perspective and a main effect of band was found. Therefore, we combined the bands and performed the post hoc test to examine the simple main effect of hand owner and perspective on the mean mu suppression at 8–13 Hz. The results confirmed that mu suppression for the own hand was stronger than for others’ hand in the first-person perspective. However, there was no significant difference in suppression between own and others’ hands in the third-person perspective.

The significant self-oriented mu suppression reactivity in the first-person perspective was found, while no significant response orientation was found in the third-person perspective. The results suggest that the reactivity of mu suppression to self and others differs depending on perspective. Nagai and Tanaka observed self and another person’s hand movements with motor imagery and demonstrated a more extensive suppression for their own hand than for another person’s hand [31]. Macuga and Frey’s fMRI study investigated brain activity contributing to self-other recognition by using videos of hands color-coded by gloves [20]. The results showed that the right inferior frontal and supramarginal gyri, constituent regions of the mirror system, were selectively activated when observing the own hand compared to the other’s hand, which might reflect processing, indicating characteristics of self-other discrimination. Based on previous findings, we predicted that the hand’s owner would affect mu suppression reactivity, regardless of the perspective, such that mu suppression would increase to the own hand in either viewpoint. However, the results showed that perspective is relevant for mu suppression, contrary to the prediction. Conson et al. examined self-other discrimination reaction times when own hand or another person’s hand picture were presented in the two perspectives. They reported that the reaction time in the third-person perspective was longer than in the first-person perspective [34]. Moreover, the relationship between the reaction time and correct answer rate differed between the perspectives. The time for detecting the self was shorter than that for detecting others in the first-person perspective, whereas the time for detecting others was shorter than that for detecting the self in the third-person perspective. However, the correct answer rate for self-identification was higher in both perspectives. The finding that responses are faster for the others’ hand in the third-person perspective, whereas the discrimination accuracy is lower than that for the own hand because the others’ hand was easily confused with the own hand, suggests the hypothesis that the self-detection system discriminates better than the others detection system. Based on this hypothesis, we speculated that the fast and accurate self-detection processing in the first-person perspective would result in a pronounced self-orientated mu suppression. However, the third-person perspective is unfamiliar because we do not usually see our hands from that perspective. The results can also be tentative interpreted as suggesting that the mu suppression discriminates between plausible and non-plausible movement. This would correspond to the mirroring of observed others actions to our motor repertoire. Further study is needed to determine whether the self-detection process or plausibility detection predominates in the expression of mu suppression.

This study indicated that lower and upper mu are unrelated to self and other differences or the perspective, although previous studies have shown that lower and upper mu have functional dissociation in the bandwidth. Focal damage to the right inferior parietal lobe, a mirror system component, selectively reduces the magnitude of mu suppression but not the upper mu range [35]. Moreover, EEG-fMRI studies of upper mu suppression have demonstrated correlations with regions engaged with the frontoparietal network associated with several cognitive processes [36]. Previous studies suggest the possibility that divided mu band responses reflect specific mirror system activities or cognitive activities associated with self-other discrimination. However, this experiment, designed to combine motor representations and self-other discrimination, might have yet to detect band-specific response stimuli.

The following limitations constrain the results of the present study. The first is whether the selection of baseline interval is appropriate. The present study used the pre-fixation baseline instead of the fixation baseline. The fixation cross is presented to stabilize measured data by focusing the gaze on a fixed point. However, in previous studies [26, 30], power suppression and rebound occurred during this fixation cross period, so the pre-fixation was selected as the baseline for stability. As a result, the mu suppression looks to differ slightly among conditions already in the segment of the fixation cross. In the statistical analysis, a two-way ANOVA with hand owner and perspective as factors for mean mu suppression during the fixation cross period (− 1 s to 0 s) showed no significant effects nor interactions (*p* > 0.1). Although there was no difference in the statistical analysis, uneven mu suppression and rebound were observed during the fixation presentation. Distinguishing mu suppression due to action observation from mu suppression due to the fixation period requires further study after considering differences in the mechanisms of their occurrence. Moreover, we did not measure self-other discrimination responses during stimulus observation to prevent artifacts. We suggest that future studies measure responses while the participants are making observations to examine the correlation more concretely between mu suppression and self-other discrimination accuracy. Furthermore, the present study suggested that mu suppression might reflect the distinction between the self and others. However, a limitation of EEG studies is that they cannot clarify whether a particular reactivity intrinsic to a neural network, such as the mirror system, generates mu suppression or whether an input external to processing generates the suppression. We expect simultaneous verification using EEG and fMRI measurements to clarify this issue.

## Conclusions

In summary, this study examined the mirror system’s activity for self and others using the EEG’s mu suppression. As a result, it was suggested that differences in self and others or perspectives may influence mu suppression.

## Supplementary Information

Supplementary Material 1.

## Data Availability

The datasets used and analyzed during the current study are available from the corresponding author upon reasonable request.

## Abbreviations

ANOVA: Analysis of variance
EEG: Electroencephalogram
fMRI: Functional magnetic resonance imaging
KSS: Karolinska Sleepiness Scale
