# Supplementary material for: Effects of observing own/others hand movement in different perspectives on mu rhythm suppression: an EEG study
Source: J Physiol Anthropol. 2024 Sep 4;43:21. doi: 10.1186/s40101-024-00369-0 (PMC11373409; doi:10.1186/s40101-024-00369-0)
Supplement: Supplementary file 1 — Supplementary Material 1. [file 40101_2024_369_MOESM1_ESM.docx]

**Effects of observing own/others hand movement in different perspectives on mu rhythm suppression: an EEG study**

Nakyeong Shin ^1, 4^, Yuki Ikeda ^2, 4^, Yuki Motomura ^3^, Shigekazu Higuchi ^3^

1. Graduate School of Integrated Frontier Sciences, Kyushu University, Japan.

2. Faculty of Health Science, Kyorin University, Japan.

3. Faculty of Design, Kyushu University, Japan.

4. Research Fellow of the Japan Society for the Promotion of Science, Japan.

Corresponding Author: Nakyeong Shin [shin.nakyeong.400@s.kyushu-u.ac.jp](mailto:shin.nakyeong.400@s.kyushu-u.ac.jp)

**Supplementary Information**

**Occipital alpha suppression.** In a result of a three-way repeated-measures analysis of variance (ANOVA) with the Band (lower, upper), Hand Owner (Self, Others) and Perspective (first-person, third-person) as the independent variables, there was a main effect of the Band (*F*(1,24) = 6.41, *p* = 0.018, *η_p_^2^* = 0.21) and there was neither a main effect of Hand Owner and Perspective, nor any interaction on alpha power suppression in occipital cluster. The differences in mu suppression activity between the conditions were not indicated for occipital alpha suppression, which is in the same frequency band as mu rhythms. Therefore, the present study results might reflect the activity and characteristics of the central region, which is somewhat separate from occipital activity.

**Supplementary Figure S1**

*Occipital alpha (8-13Hz) power suppression*

*
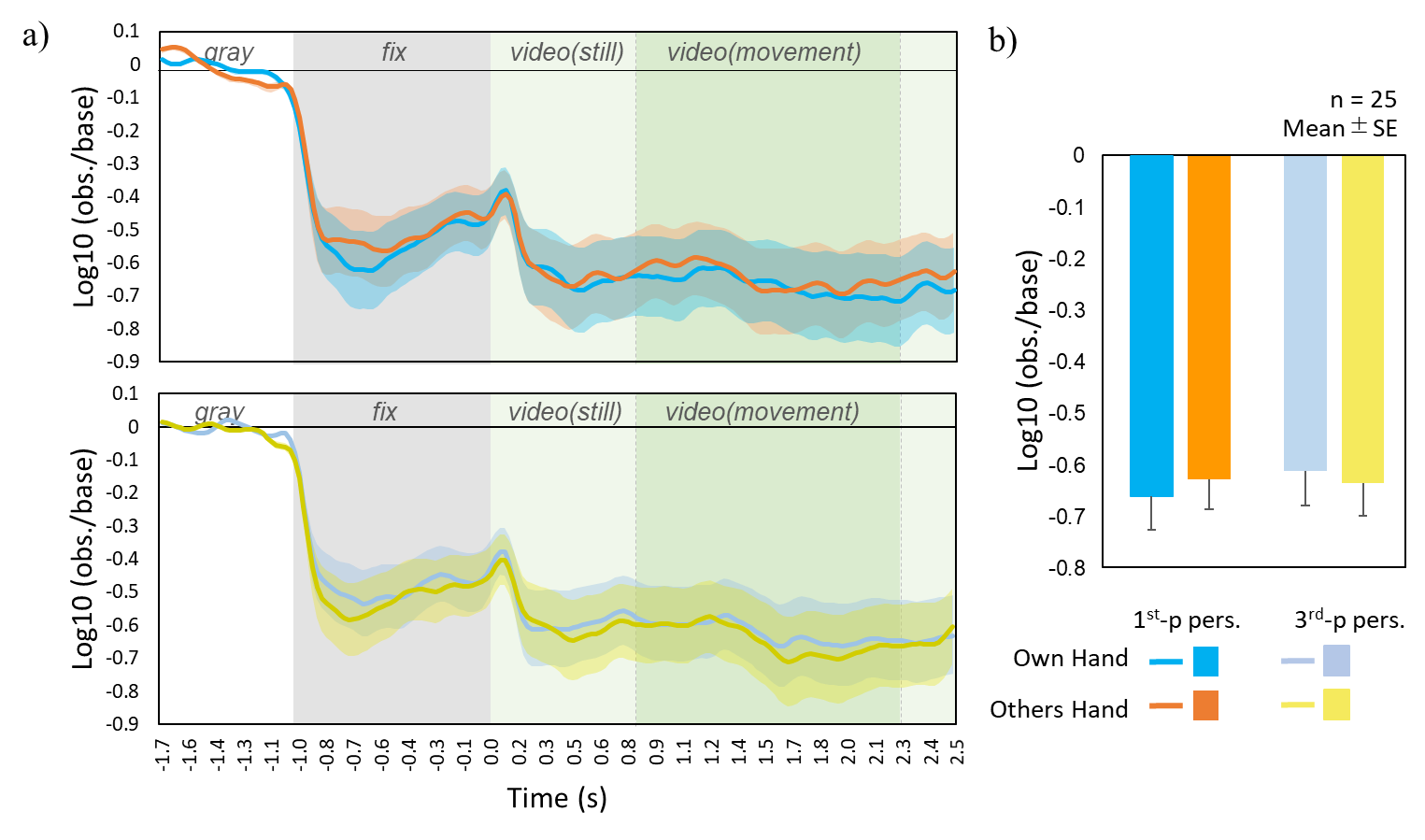
*

*Note.* (a)The log10 power ratio’s time course for each condition. The elements within an epoch (gray background as the baseline, the fixation cross, and the video clip including still and moving parts) are labeled in different colors. (b) Mean (±SE) log10 power ratio when observing the moving part of video clip under different conditions.

**Lower/Upper mu suppression.** A three-way repeated ANOVA showed a significant main effect of the lower and upper bands in mu suppression (*F*(1,24) = 7.099, *p* = 0.013, *η_p_^2^* = 0.23), indicating a more potent overall suppression in the lower mu band compared to the upper mu band. There were no significant interactions involving Band. The observed main effect of the Band indicates that there are differences in the magnitude of suppression between the bands. However, the fact that no interaction was observed suggests that these differences in bandwidth are not influenced by the factors of Hand Owner or Perspective.

**Supplementary Figure S2**

*Lower (8-10Hz) and Upper(10-13Hz) mu power suppression.*

*
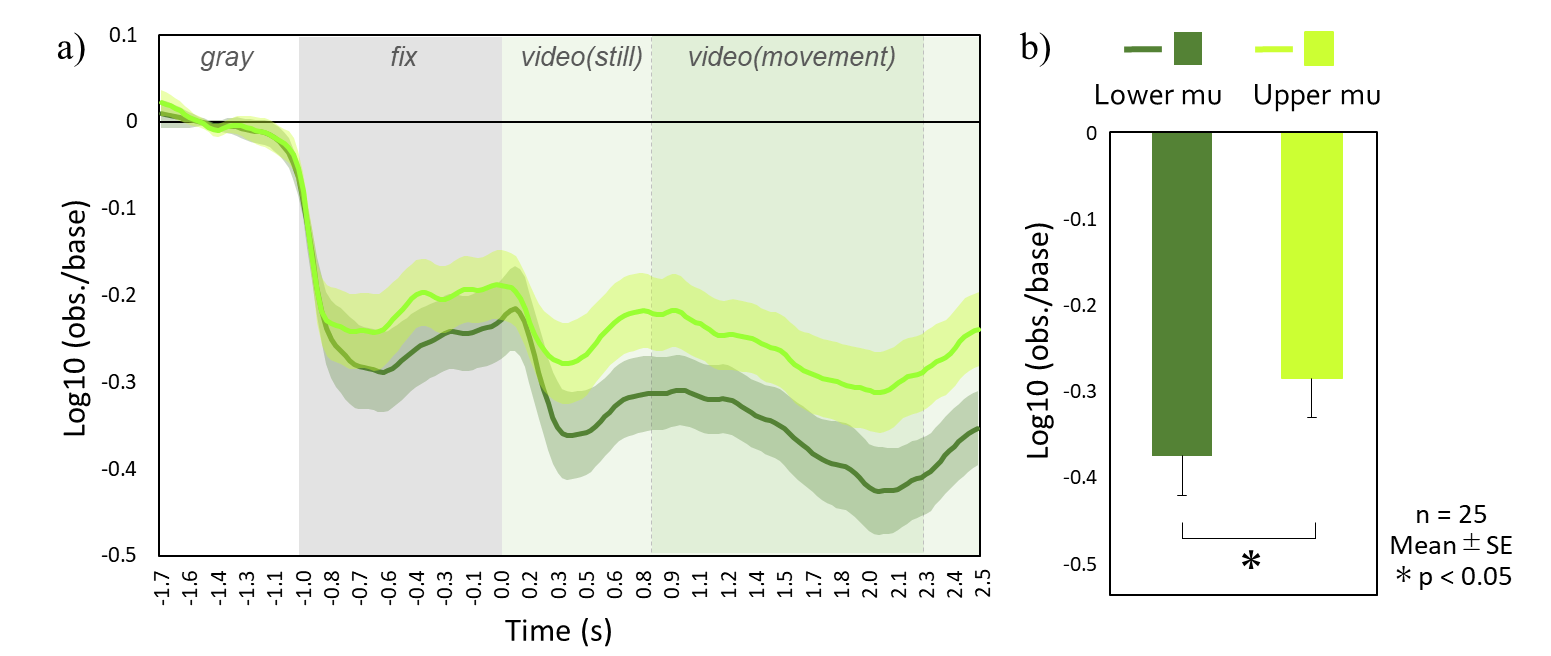
*

*Note.* (a)The log10 power ratio’s time course for each condition. The elements within an epoch (gray background as the baseline, the fixation cross, and the video clip including still and moving parts) are labeled in different colors. (b) Mean (±SE) log10 power ratio when observing the moving part of video clip under different conditions.
